# Aromatic Medicinal Plants from Tajikistan (Central Asia)

**DOI:** 10.3390/medicines2010028

**Published:** 2015-02-17

**Authors:** Farukh S. Sharopov, Hanjing Zhang, Michael Wink, William N. Setzer

**Affiliations:** 1 Institute of Pharmacy and Molecular Biotechnology, Heidelberg University, Im Neuenheimer Feld 364, 69120 Heidelberg, Germany; 2 V. I. Nikitin Institute of Chemistry, Tajik Academy of Sciences, Ainy St. 299/2, Dushanbe 734063, Tajikistan; 3 Department of Chemistry, University of Alabama in Huntsville, Huntsville, AL 35899, USA; E-Mail: hanjing228@gmail.com

**Keywords:** Tajikistan, aromatic plants, traditional herbal medicines, essential oil compositions, secondary metabolites

## Abstract

Tajikistan is a small country located in Central Asia. The mostly mountainous terrain with a continental, subtropical, and semiarid climate, is characterized by diverse flora. Many people in Tajikistan rely on medicinal plants as their traditional form of medicine to prevent and cure health disorders. Aromatic medicinal plants, in particular, have played an important role for the local people. In this review, we present a summary of the uses of 18 aromatic medicinal plants from Tajikistan and their compositions of secondary metabolites.

## 1. Introduction

Plants have been and continue to be valuable natural treasures, providing an important source of nutrients and therapeutic agents. Plants must defend themselves against herbivory and microbial infections, and over the last 400 million years they have evolved a high diversity of secondary metabolites that are toxic to animals and microorganisms. Because of this evolutionary background, most secondary metabolites are biologically active. This did not escape our ancestors who started to use plants as a means to treat infections and health problems [1,2]. In this context, traditional medicine and our understanding of the pharmacological properties of plants were developed.

The defense chemistry of plants includes a surprisingly wide diversity of biologically active compounds, such as alkaloids, glucosinolates, cyanogenic glycosides, flavonoids, tannins, coumarins, lignans, terpenoids, saponins, organic acids, and many others. A number of secondary metabolites, especially mono- and sesquiterpenes but also phenylpropanoids, are volatile. These compounds serve not only as deterrents against herbivory and often against microbial infection, but also as signal compounds to attract pollinators or predators. Aromatic plants have attracted human attention for a long time because of their mostly pleasant fragrances. As a consequence, many of them are used as raw materials for the production of perfumes and cosmetics; others have found application in aromatherapy and phytotherapy. Many aromatic plants serve as spices because they can reduce the load of microbial pathogens in food, improve the taste, and support digestion (e.g., as carminatives and choleretics).

**Figure 1:**
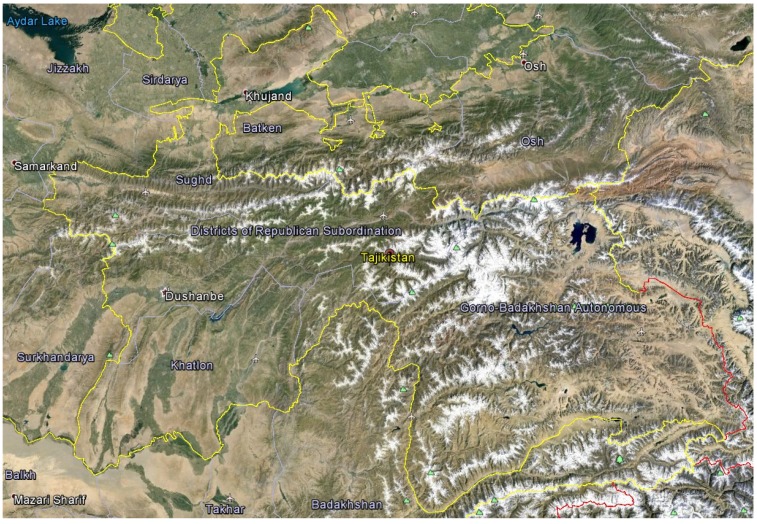
Google Earth^©^ satellite view of Tajikistan with provincial boundaries.

Tajikistan is a mountainous country in the southeastern part of Central Asia (mostly between 36° and 41° N latitude, and 67° and 75° E longitude. Tajikistan shares borders to the north and northwest with Uzbekistan and Kyrgyzstan, to the south with Afghanistan, and with China to the east. The country covers 143,100 km^2^ (93% of this area is mountainous) and has a population of approximately eight million people. Tajikistan is divided into four provinces (viloyat). These are the provinces of Sughd and Khatlon, the autonomous province of Gorno Badakhshan, and the Districts of Republican Subordination (Figure 1). Tajikistan is diverse in terms of environmental conditions including climate, high altitudes, mountainous soil and minerals, relatively large number of sunny days per year, which can all affect plant growth, biosynthesis, and accumulation of biological active secondary metabolites. High mountains dominate the country with about 50% above 3000 m above sea level, and the relief differentiation has resulted in formation of numerous micro- and macrohabitats. The elevation starts at 300 m above sea level and ends with Ismoili Somoni Peak at 7495 m. There are over 900 rivers in Tajikistan longer than 10 km. The high-mountain ecosystems of Tajikistan have been regarded as biodiversity hotspots with around 4550 species of higher plants recorded in Tajikistan and about 30% endemism [3,4]. The high degree of biodiversity and endemism in Tajikistan is due to the presence of high mountain ranges that serve as barriers to migration of plants and animals. Additionally, Tajikistan is characterized by a low percentage of cloud cover, large temperature differences (absolute minimum of −63 °C in the Pamir and maximum of 48 °C in Panji Poyon), low humidity, and low precipitation. Several altitudinal plant zones have been described [3,5]: (1) sage desert dominated by *Artemisia* species; (2) juniper woodlands; (3) desert steppes; (4) high mountain deserts dominated by cushion plants; and (5) alpine.

Many Tajik plants have been used since ancient times in traditional medicine. The scientist Abu Ali ibn Sina (Avicenna) (born 980 in Afschana near Buchara in what is now Uzbekistan; died 1037 in Hamadan) described more than 750 pharmaceutical substances of vegetative, animal, and mineral origin, several of them from Central Asia, in his book Al-Qanoon fi al-Tibb (The Canon of Medicine). Many medicines (drugs) described by Avicenna have entered the pharmacopeia and are still in use [6]. His experience was influenced from his years in Central Asia, but he was also aware of the important *Materia Medica* of Dioscorides (who had lived 900 years earlier) [1,2]. The *Materia Medica* of Avicenna comprised more than 50 cardiac, 70 antiasthmatic, and 75 antidiabetic plants; 110 plants were described as useful for the treatment of kidney and gallstones, more than 40 plants for the treatment of vitiligo, dozens for wound healing, and others with antitoxic, antitumor, hemostatic and several other activities [7].

Medicinal plants generally contain complex mixtures of biologically active compounds. They affect multiple targets and, in general, show low toxicity. Some of the active secondary metabolites can have advantages in treating chronic diseases. In aromatic plants, part of their therapeutic effects comes from their essential oils. Most secondary metabolites in essential oils are small lipophilic natural products, which allow them to readily enter body tissues by free diffusion. The lipophilic components can interact with biomembranes and membrane proteins. They thus influence membrane fluidity and permeability. This explains why many components of essential oils exhibit antibacterial, antifungal, antiviral, and cytotoxic activities [8].

Tajikistan has a rich flora including large numbers of herbs and aromatic plants. According to preliminary estimates, about 1500 species of Tajik plants are used in folk medicine, but only a small number of them are important in modern medicine [9]. In this review we have focused on the ethnopharmacology and phytochemistry of a number of aromatic medicinal plants from Tajikistan, with emphasis on the compositions and biological activities of their essential oils.

## 2. Tajik Aromatic Medicinal Plants

### 2.1. Achillea filipendulina Lam., “Yarrow”, “Buimodaron” (Asteraceae)

A number of *Achillea* species have been used in folk medicine in Europe and Asia [10]. *A. filipendulina* has been employed since ancient times in traditional herbal medicines against a variety of ailments [11]. According to the “Canon of Medicine”, decoctions of *A. filipendulina* have been used to treat “breaking the muscles” and chronic inflammation of the sciatic nerve (sciatica). In Tajik folk medicine, a decoction from the dried flowers of *A. filipendulina* is applied as a children’s digestive aid, to treat stomach ache and cough [12,13]. This plant is also traditionally used as an emmenagogue and expectorant [14], and has been used externally to treat scabies and wounds [15]. The essential oil of Tajik *A. filipendulina* is rich in santolina alcohol **(1)** (43%–46%), 1,8-cineole **(2)** (9%–11%), borneol **(3)** (5%–6%), isoborneol (5%) and *cis*-chrysanthenyl acetate **(4)** (7%–9%) [16]. 1,8-Cineole has a number of biological activities that make it particularly useful in the treatment of respiratory tract infections [17]. *A.* *filipendulina* leaf oil from Iran has shown antibacterial activity [15]. The major flavonoids from the leaf exudates of *A. filipendulina* are quercetagetin and centaureidin [18]. Quercetagetin has shown anti-HIV activity (inhibitor of HIV reverse transcriptase [19] and HIV integrase [20]), while centaureidin has shown cytotoxic activity (tubulin polymerization inhibitor) [21].

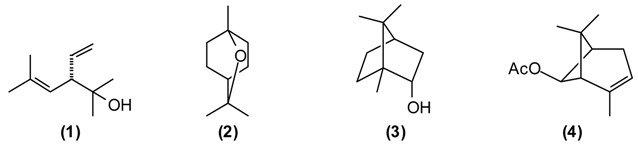


### 2.2. Anethum graveolens L., “Dill”, “Shibit” (Apiaceae)

This plant is widely used in Europe and Asia for flavoring foods and beverages due to its pleasant spicy aroma. It has been extensively utilized as a traditional herbal medicine throughout Europe, Asia, and America. In Tajik folk medicine, water extracts (tea and infusion) of *A. graveolens* are widely applied for improving appetite, treating flatulence, stomach problems, digestive disorders, insomnia, cramps, inflammations of the respiratory tract, and for stimulating the release of milk in nursing mothers [13,14]. The major components of *A. graveolens* essential oil from Tajikistan are carvone **(5)** (52%), *trans*-dihydrocarvone (15%), dill ether **(6)** (13%), α-phellandrene **(7)** (8%), and limonene **(8)** (7%) [22].

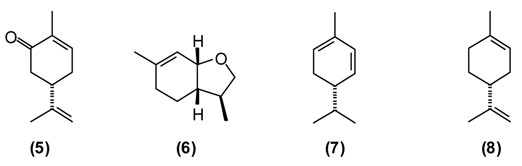


The cytotoxicity of the oil was assessed against human cervical cancer (HeLa), human colorectal adenocarcinoma (Caco-2) and human breast adenocarcinoma (MCF-7) cancer cell lines. IC_50_ values were 0.093 mg/mL for HeLa, 0.216 mg/mL for Caco-2, and 0.067 mg/mL MCF-7 cell lines. *A. graveolens* essential oil was toxic in the brine shrimp lethality test (LC_50_ = 15.9 μg/mL), but showed only marginally antimicrobial activity against *Escherichia coli* (MIC = 625 μg/mL) [22]. The bioactivity of *A. graveolens* oil is most likely due to the major components carvone and limonene. Both (*R*)-(+)-limonene and (*S*)-(+)-carvone increase the production of reactive oxygen species (ROS) and decrease mitochondrial membrane potentials (MMP) [23]. Carvone is added to toothpastes, mouthwashes, and chewing gums. It is also used as a taste enhancer in the food and fragrance industry. Due to its spasmolytic effect, (*S*)-(+)-carvone is utilized as a stomachic, carminative, and for treatment of nervous tension and several skin disorders [24].

### 2.3. Artemisia absinthium L., “Wormwood”, “Tkhach” (Asteraceae)

Wormwood is a traditional medicinal plant in Europe and Asia. It was widely used in the alcoholic beverage “absinthe” that was later banned in many countries because of its neurotoxicity, which is thought to be due to the presence of thujone. [1]. *A. absinthium* is an herb also used traditionally in Tajikistan. This plant is known to possess several biological properties, especially anthelmintic, digestive, antifungal, antibacterial, but also balsamic, diuretic, and emmenagogue activities. Extract of wormwood (~2 teaspoons chopped herbs in a glass of boiled water, the daily dose) is utilized to treat hyperacidity, gastric colic, gastritis, flatulence, and conditions of the liver and gallbladder. Chronic, large doses of wormwood have been reported to upset the nervous system [11,12,25]. The major components of *A. absinthium* oil from Tajikistan are myrcene **(9)** (9%–23%), *cis*-chrysanthenyl acetate **(4)** (8%–18%), a dihydrochamazulene isomer (6%–12%), germacrene D **(10)** (2%–8%), linalool **(11)** (5%–7%), and β-thujone **(12)** (up to 7%), and is phytochemically distinct from *A. absinthium* from Europe, the Middle East, or Siberia [26].

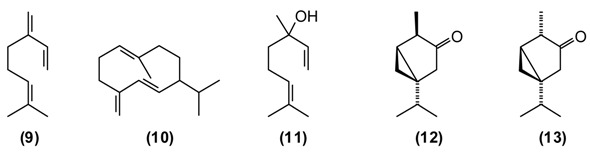


Antifungal, antimicrobial activity, choleretic, antiseptic and balsamic properties of plant can be explained by the composition of its essential oil. Myrcene-containing *A. absinthium* essential oil is used in the manufacture of alcoholic beverages and in pharmaceutical preparations as a mild sedative in the treatment of insomnia [24].

### 2.4. Artemisia rutifolia Stephan ex Spreng., “Poosh” (Asteraceae)

This plant is used as a tonic, febrifuge and anthelmintic in traditional medicine. A powder of the plant mixed with honey is useful against worms. A tea prepared from the dried and chopped herb is drunk to treat asthma, weakness of the heart, and also as anti-inflammatory, diuretic, and anthelmintic [6,7,12]. *A. rutifolia* from Tajikistan belongs to the thujone-rich chemotype, in contrast to the cineole/camphor chemotype found in Mongolia, and is dominated by α-thujone **(12)** (21%–37%), and β-thujone **(13)** (36%–47%), as well as 1,8-cineole **(2)** (3%–12%), and germacrene D **(10)** (2%–3%) [27].

The bioactivity of *A. rutifolia* is most likely due to the thujones that are present in its essential oil. However, thujone has psychotropic effects, acting on the γ-aminobutyric acid-gated chloride channel, a member of the superfamily of ligand-gated ion channel receptors [28]. In addition to the essential oil, *A. rutifolia* is rich in guaianolide, germacranolide, and eudesmanolide sesquiterpenoids [29,30,31]. In general, α-methylene lactones have shown potent antitumor, antischistosomal, anthelmintic, and antimicrobial properties [32,33].

### 2.5. Artemisia scoparia Waldst & Kit., “Joroob” (Asteraceae)

In the folk medicine of Tajikistan, decoctions and infusions from the tops of the shoots of *A. scoparia* are used to treat kidney disorders, as well as a diaphoretic, diuretic, and anthelmintic. Decoctions of the plant are considered useful against epilepsy, rheumatism, fever, and inflammation of the respiratory tract [11]. According to some authors [12,25] the aerial parts of *A. scoparia* are useful as an expectorant. *A. scoparia* essential oils from different geographical locations exhibit a great variability, but that from Tajikistan is dominated by the diacetylenes 1-phenyl-2,4-pentadiyne (34%) and capillene **(14)** (5%), as well as β-pinene **(15)** (21%), α-pinene **(16)** (5%), methyl eugenol **(17)** (6%), myrcene **(9)** (5%), limonene **(8)** (5%), and (*E*)-β-ocimene (4%) [34].

Polyacetylenes from plants are known to be highly toxic against fungi, bacteria, and mammalian cells, and to display neurotoxic, anti-inflammatory and anti-platelet-aggregation effects and to be responsible for allergic skin reactions [35]. Because of the reactive triple bonds they can alkylate a variety of proteins, thus changing their activity [8]. β-Pinene has also shown antimicrobial activity [36]. 
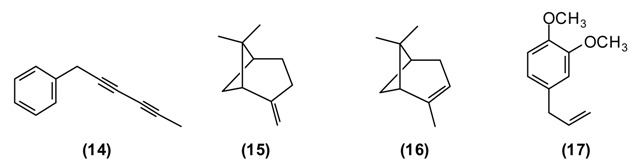


### 2.6. Bunium persicum B. Fedtsch., “Black Cumin”, “Zira” (Apiaceae)

*B. persicum* is extensively used in traditional medicine to treat chronic diseases of the stomach (chronic gastritis), intestines (colitis), liver (jaundice), chronic cholangitis, swelling, and also against kidney stones. Tea from *B. persicum* seeds is considered a popular means to increase the appetite. According to Sakhobiddinov [37], the fruit of zira is useful against stomach ache and to treat an enlarged spleen. Infusion of the fruits stops nosebleeds, while the roasted fruits are considered as a diuretic. In rural/village traditional medicine, zira strengthens the stomach and intestines, stimulates the appetite, eliminates flatulence, and drives the urine. Zira enhances wound healing and continuous use of zira is thought to prevent obesity. In addition, the plant is used widely as a condiment for culinary purposes and flavoring foods and beverages [11,14]. *B. persicum* essential oil has demonstrated antispasmodic activity in support of its traditional therapeutic use of the plant against gastrointestinal disorders [38]. Oil from seeds of the Tajik *B. persicum* is composed largely of cuminaldehyde **(18)** (36%–37%), γ-terpinen-7-al **(19)** (15%–17%), α-terpinen-7-al **(20)** (13%), γ-terpinene **(21)** (10%–11%), β-pinene **(16)** (9%), and *p*-cymene (5%). The composition of wild-growing *B. persicum* from Tajikistan is comparable to that found in commercial *B. persicum* from India: cuminaldehyde (30%), γ-terpinen-7-al (17%), α-terpinen-7-al (8%), γ-terpinene (11%), β-pinene (8%), anthemol (10%), and *p*-cymene (13%). 
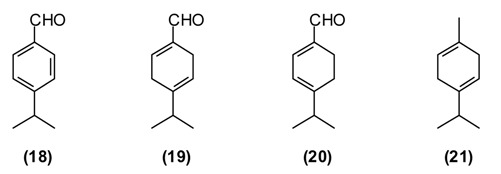


The carbonyl group of aldehydes is very electrophilic and can react with a variety of nucleophiles such as amino groups either from proteins and nucleic acids [39,40]. Cuminaldehyde has shown antibacterial [41,42] and antifungal [43] activity.

### 2.7. Galagania fragrantissima Lypsky, “Shnk”, “Shibite” (Apiaceae)

This plant is distributed in Afghanistan, Kyrgyzstan, Uzbekistan and Tajikistan. The leaves and young shoots are used as a spice for soups and other dishes [44]. The main constituents of the essential oil of *G. fragrantissima* are a series of aliphatic aldehydes and alcohols such as (2*E*)-dodecenal **(22)** (84%), (2*E*)-dodecenol **(23)** (8%), (2*E*)-tetradecenal **(24)** (3%) and dodecanal **(25)** (2%).

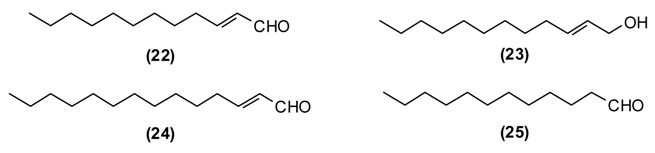


*G. fragrantissima* oil exhibits cytotoxicity in HeLa, Caco-2 and MCF-7 cancer cell lines: IC_50_ values were 0.206 mg/mL for HeLa, 0.074 mg/mL for Caco-2, and 0.058 mg/mL for MCF-7 cell lines [45]. The activity of this essential oil can be attributed to the long-chain aldehydes. Biological activities of (2*E*)-dodecenal **(22)**, (2*E*)-tetradecenal **(24)** and dodecanal **(25)** are correlated to physico-chemical damage to the cells, such as the disruption of the membrane and probably interference with proteins [39] and nucleic acids [40]. Additionally, due to the structural similarities to fatty acids, aliphatic aldehydes (dodecenal, dodecanal and tetradecenal) inhibit 5-lipoxygenase, a key enzyme in inflammatory processes [39].

### 2.8. Hypericum perforatum L., “St. John’s Wort”, “Choykah” (Hypericaceae)

St. John’s wort is a traditional medicinal plant in Europe and Asia. In modern phytotherapy, special extracts are employed with proven efficacy against depression [1,2], possibly acting by several mechanisms: as a blocker of serotonin, noradrenaline and dopamine reuptake; causing increase in serotonergic and dopaminergic receptor density and increased affinity for GABAergic receptors; and inhibition of monoaminoxidase activity [46,47]. The antidepressant activity of *H. perforatum* has been linked to the presence of hypericin and pseudohypericin [48], along with hyperforin [49]. In folk medicine, *H. perforatum* is widely used in the treatment of gallbladder conditions and cystitis, chronic gastritis, gastric ulcer and duodenal ulcers, wounds, burns, gout and rheumatism, to strengthen the gums and alleviate halitosis, and also to treat involuntary urination in children. *H. perforatum* is used as a wound-healing aid in France [50] and in Turkey [51]. Flavonoids were found to be the active components [52]. The people in Russia called *H. perforatum* “means for the ninety-nine disease.” According to Russian national doctors, “As without flour it is impossible to bake bread, so without *H. perforatum* it is impossible to treat many illnesses of people and animals.” [14]. *H. perforatum* stimulates appetite, improves bowel, enhances diuresis, and has a styptic and general calming effect. Its tincture has a beneficial effect against chronic gastritis [11]. In a rat model of inflammatory bowel disease, *H. perforatum* extract reduced colonic damage, attributable to anti-inflammatory and antioxidant effects [53,54]. There are numerous chemotypes of *H. perforatum* based on essential oil composition and geographical location [55]. Tajik *H. perforatum* oil was characterized by germacrene D **(10)** (14%) as a major secondary metabolite, α-pinene **(16)** (5%), β-caryophyllene **(26)** (5%), caryophyllene oxide **(27)** (4%), bicyclogermacrene **(28)** (4%), dodecanol (5%), and spathulenol **(29)** (3%) [55].

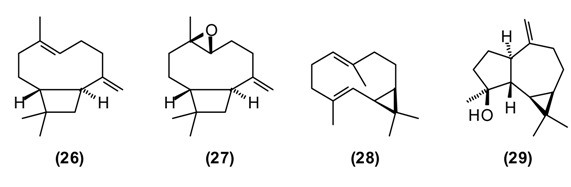


Germacrene D has shown antifungal activity against *Aspergillus niger* as well as cytotoxic activity in PC-3 cells [56]. β-Caryophyllene exhibits several biological properties including anti-inflammatory, antibiotic, antioxidant, anticarcinogenic and local anaesthetic activities. It has a potentiating effect on the anticancer activity of several compounds [57]. Caryophyllene oxide shows *in vitro* cytotoxicity against MCF-7, PC-3, and Hep-G2 cells [56].

### 2.9. Hypericum scabrum L., “Choykah” (Hypericaceae)

*H. scabrum* is used in traditional medicine to treat a variety of disorders of liver, heart, stomach, intestines, bladder, cough, *etc.* A “marham” (poultice made from the herb and butter) is applied externally to treat sores, ulcers, abscesses, furuncles, and mastitis. An infusion of the flowers is recommended against jaundice [7,11]. The essential oil of *H. scabrum* from Tajikistan is dominated by α-pinene **(16)** (45%), with lesser amounts of spathulenol **(29)** (7%), verbenone **(30)** (6%), *trans*-verbenol **(31)** (4%), and γ-muurolene **(32)** (4%) [55]. Both *H. scabrum* essential oil [58] and methanol extracts [59] have shown antimicrobial activity. Interestingly, the anti-inflammatory and chondroprotective activity of (+)-α-pinene is believed to be greater than that of (–)-α-pinene or β-pinene [60]. 
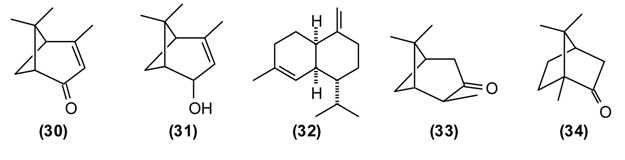


### 2.10. Hyssopus seravschanicus Pazij, “Hyssop”, “Ushnondoru” (Lamiaceae)

This plant is a perennial semi-shrub that can be found growing wild in the northwestern part of Tajikistan. Hyssop has been used in Tajik folk medicine for centuries. Avicenna recommended hyssop for its antiseptic, anti-inflammatory, wound healing, analgesic, antitussive and stimulating activities [61]. Decoctions of hyssop are used to treat bronchial asthma, chronic bronchitis, flu and diseases of the respiratory tract. Additionally, it is used to relieve inflammation of the urinary tract.

The volatile compounds from *H. seravschanicus* growing wild near the high ridge mountainous regions of Varzob, Northern Dushanbe in Tajikistan at an altitude approximately 2500 m above sea level, were extracted and analyzed using gas-liquid chromatography-mass spectrometry (GLC-MS). The most abundant components were *cis*-pinocamphone **(33)** (57%–89%), β-pinene **(15)** (up to 6%), 1,8-cineole **(2)** (2%–4%), camphor **(34)** (up to 4%), and spathulenol **(29)** (up to 5%). The essential oil from *H. seravschanicus* showed low antibacterial activity against *Bacillus cereus* and *Staphylococcus aureus* [62], likely due to the high concentration of *cis*-pinocamphone [63,64].

### 2.11. Melissa officinalis L., “Lemon Balm”, “Niyozbuy” (Lamiaceae)

Lemon balm has been used in Europe and Asia to reduce stress and anxiety, promote sleep, improve appetite, and ease pain and discomfort from indigestion [65]. Avicenna believed that lemon balm refreshes and strengthens the heart, helps in digestion and hiccup. He recommended lemon balm as a tonic and for the treatment of melancholia [61]. Lemon balm leaves have long been used as anxiolytics, mood elevators and a calming herb for patients with anxiety [66]. The plant is also used as an additive in food, for the production of many phytopharmaceutical preparations, fragrances and cosmetics [67].

The main constituents of the essential oils of *Melissa officinalis* from Tajikistan were geranial **(35)** (43%), neral **(36)** (31.5%), *trans*-anethole **(37)** (12%), β-caryophyllene **(26)** (4%) and citronellal **(38)** (3%). *Melissa officinalis* essential oil was cytotoxic to MCF-7 cells (IC_50_ = 0.062 mg/mL) and active in the brine shrimp lethality test (LC_50_ = 21.8 μg/mL), but showed only marginal antimicrobial activity against *Bacillus cereus* (MIC = 313 μg/mL) and *Aspergillus niger* (MIC = 625 μg/mL) [68]. The biological activities can be attributed to the monoterpenoid aldehydes, which can form Schiff’s bases with free amino groups of peptides and proteins, thus changing their biological properties [8]. Citral, a mixture of geranial and neral, has shown *in vitro* cytotoxic activity [69]. Citral has also been found to inhibit contractions of rat ileum [70], while geraniol has shown smooth muscle relaxant activity [71]. Lemon balm leaves are rich in the antioxidant rosmarinic acid, which has antiviral properties and was used to treat herpes infections [1,2].

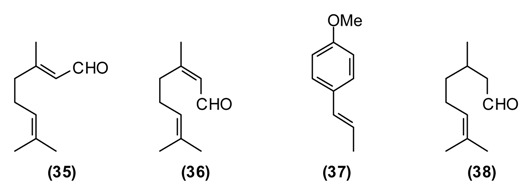


### 2.12. Mentha longifolia (L.) Huds., “Wild Mint”, “Hulba, Pudina” (Lamiaceae)

*Mentha* species have been in use by humans since ancient times in many parts of the world [2]. The aerial parts of *Mentha* species are commonly used in many processed foods as well as in herbal teas. In folk medicine, mint is widely utilized as a tea or as a gargle against both acute and chronic conditions of the upper respiratory tract. It is prescribed against liver disorders (jaundice, chronic hepatitis), intestinal spasms, biliary tract infections (acute and chronic cholecystitis), and cholangitis [11,14]. Mint juice from freshly picked leaves as well as dried and crushed mint leaves are widely used to improve appetite and digestion, as anti-inflammatory, diaphoretic, carminative, antiemetic, antitussive, and analgesic agents. Infusions of mint are employed to prepare washes and lotions for treating spasms, rheumatic pains, arthritis, itching and inflammation of the skin [72]. Mint is also used to repel insects, snakes, and worms [11]. Mountain mint has been used externally to treat cracks in the skin caused by dry skin as well as bone fractures and internally to relieve muscular aches and sciatica. A compress treated with a hydroalcoholic extract mountain mint is used to treat bruises and animal bites [14]. Chemical compositions of *M. longifolia* essential oils from different geographical locations varies considerably, even within Tajikistan [73]. Wild populations of Tajik *M. longifolia* can be dominated by *cis*-piperitone epoxide **(39)** (up to 78%), piperitenone oxide **(40)** (up to 49%), carvone **(5)** (up to 22%), menthone **(41)** (up to 17%), as well as pulegone **(42)** (1%–5%) and thymol (2%–4%) [73]. The essential oil has shown moderate antimicrobial activity, supporting the traditional use of this plant to treat wounds and skin infections [74].

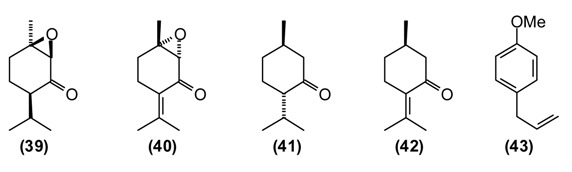


### 2.13. Ocimum basilicum L., “Basil”, “Rayhon” (Lamiaceae)

Basil is a popular plant cultivated in Europe and Asia, but also in Tajikistan and used frequently to flavor soups, desserts, pickles, pizza, spaghetti sauce, tomato juice, salads, *etc.* The plant is widely used in food and oral care products and well known in folk medicine. In Tajik folk medicine, the water extract of basil is used to treat inflammatory diseases of the upper respiratory tract (bronchitis, laryngitis, pharyngitis, *etc.*), chronic gastritis, enterocolitis, and food poisoning. Additionally, hot basil tea is taken to treat nausea, flatulence, and dysentery. The volatiles of basil are known to repel flies [75], mosquitoes [76], and other insects [77]. The essential oil of Tajik basil is dominated by linalool **(11)** (47%), methylchavicol (estragole) **(43)** (32%), and pulegone **(42)** (5%). The major components in basil oil, linalool and methylchavicol, have shown anti-inflammatory activities [78], supporting the rationale for the basil traditional use in inflammatory diseases of the upper respiratory tract. Linalool has also shown antibacterial [79] and antiviral [80] activities.

### 2.14. Origanum tyttanthum Gontsch. “Kokuti”, “Sebinak” (Lamiaceae)

In Tajikistan, where *O. tyttanthum* is a common species, this plant has commercial value. Cultivation of *O. tyttanthum* covers a total area of over 140,000 hectares, yielding annually a total of 6490 tons of air-dried raw materials [81]. As a medicinal plant, *O. tyttanthum* has traditionally been used as an expectorant, carminative, diaphoretic, stimulant, stomachic, and tonic. In addition, it has been used as a folk remedy against colic, coughs, headaches, nervousness, toothaches, and irregular menstrual cycles. Teas prepared from the aerial parts of *O. tyttanthum* have been used to treat tuberculosis and against human intestinal parasites. They are also used for sedative purposes and widely used against flatulence and as a gargle against laryngitis, stomatitis, and angina [11,14,37,82]. The major components of the Tajik essential oil are carvacrol **(44)** (34%–59%), thymol **(45)** (11%–46%), and *p*-cymene **(46)** (1%–7%) [83]. The ointment “Subinak” was created on the basis of the essential oil of *O. tyttanthum* from Tajikistan [9]. The presence of phenolic monoterpenes (carvacrol and thymol) with known antiseptic properties [84] as the major oil components is responsible for its potent antioxidant, antibacterial, fungicidal, insecticidal, herbicidal, and nematicidal activities [85]. 
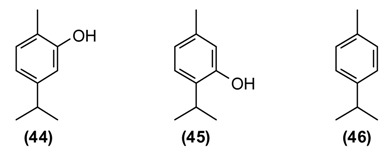


### 2.15. Pelargonium graveolens L'Hér. “Rose-Scented Geranium”, “Anjibar” (Geraniaceae)

*Pelargonium graveolens* L. Her. *ex* Ait. (synonym *P. roseum* Willd.) was introduced into Tajikistan approximately 80 years ago, and it does not have a traditional medicine background. In Tajikistan, it is cultivated for the production essential oil for perfumery and as a decorative ornamental plant. *P. graveolens* oil is a recommended aromatherapy for anxiety and depression, and the oil has demonstrated anxiety reduction after inhalation [66]. The Tajik essential oil of *P. graveolens* is dominated by citronellol **(47)** (38%), lower amounts of geraniol **(48)** (6%) and linalool **(11)** (3%), along with numerous citronellol and geraniol esters (10% and 7%, respectively) [86,87]. 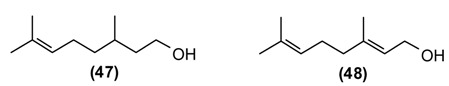


### 2.16. Salvia sclarea L. “Clary Sage”, “Marmarak” (Lamiaceae)

Clary sage is a traditional medicinal plant in Europe and Asia [2]. This plant has been used in Tajikistan as a folk remedy for the treatment at palpitation, for improvement of digestion, against colds and throat disturbances, and also as a tonic against fatigue. An infusion from the aerial parts is employed to treat conditions of the kidney and to reduce fever. A tea prepared from the aerial parts of *S. sclarea* is taken to improve digestion and appetite, and also as a diuretic. *S. sclarea* fruits are utilized to treat dysentery and bloody diarrhea. *S. sclarea* is applied externally to soften the skin [11,14,82]. Commercial grade clary sage oil is rich in linalyl acetate **(49)** and linalool **(11)**, and Tajik oil is comparable with 39% linalyl acetate and 13% linalool, as well as germacrene D **(10)** (11%), α-terpineol **(50)** (6%), geranyl acetate (4%), and β-caryophyllene (2%) [88]. Plants from the genus *Salvia* are a rich source of polyphenols (more than 160 polyphenols have been identified in *Salvia*) which are believed to be responsible for the many biological activities of sage and their use in traditional medicine [64]. Small lipophilic molecules of the essential oil, which interfere with biomembranes of microbes, are thought to be helpful against colds, fever, throat, and kidney conditions.

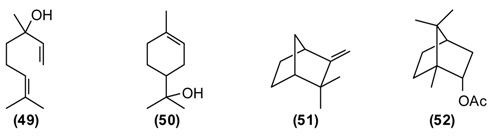


### 2.17. Tanacetum parthenium L. Schultz-Bip., “Feverfew”, “Bobunai Govi” (Asteraceae)

Feverfew is a traditional medicinal plant in Europa and Asia [2]. Aerial parts of *T. parthenium* have been used for the treatment of migraine, arthritis, and problems associated with menstrual cycle. Parthenolide [89] and tanetin [90] are the principal non-volatile active chemicals of *T. parthenium*. The essential oil of *T. parthenium* from Tajikistan revealed only eight components. The major components were camphor **(34)** (70%–94%), camphene **(51)** (2%–12%), and bornyl acetate **(52)** (4%–9%). [91]. The essential oil has antioxidant (DPPH and ABTS radical scavenging, lipid peroxidation inhibition), cytotoxic (HeLa, CCRF-CEM, and CEM/ADR5000 cell lines), and lipoxygenase inhibitory activities. The cytotoxicity of feverfew oil can be attributed to the monoterpenoids, which are lipophilic and can dissolve in biomembranes, disturbing fluidity and permeability [8,40].

### 2.18. Ziziphora clinopodioides Lam. “Blue Mint”, “Jamilak” (Lamiaceae)

*Z. clinopodioides* is an edible medicinal plant that is widely distributed in Tajikistan. The leaves, flowers and stem of the plant are frequently used as a wild vegetable or as an additive to foods. The plant has been used since ancient times in traditional herbal medicines for the treatment of colds and cough, stomach ache, nausea, poor appetite, sexually transmitted diseases, and as an antiseptic and to promote wound healing [11,12,14]. Three chemotypes of *Z. clinopodioides* have been recognized according to the composition of the essential oil, namely pulegone-rich, thymol-rich, and cineole-rich chemotypes [92]. Tajik *Z. clinopodioides* oil is a pulegone-rich oil, composed mainly of pulegone **(42)** (73%–35%), neomenthol **(53)** (7%–23%), and menthone **(41)** (6%–13%) [93]. Pulegone-rich essential oils have shown antiviral activity [93]. Pulegone can irritate mucosal tissues of the GI-tract and externally the skin. It can cause spasms and cramps [1]. Like most essential oils, *Z. clinopodioides* oil has lipophilic properties, targeting biomembranes, which would explain its antiseptic properties [8]. 
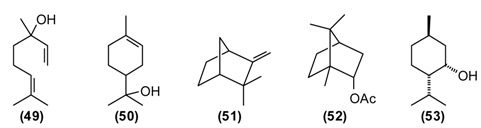


## 3. Conclusions

The rich and diverse flora of Tajikistan provides numerous locally available plants useful in traditional herbal medicine, among them Tajik aromatic medicinal plants with bioactive essential oils. Many of them have shown antibacterial, antiviral, antioxidant, and cytotoxic bioactivities. The essential oils play a commercial role as flavors, fragrances, cosmetics, and pesticides. In addition, they are also used to prevent and treat infections and other human health disorders, including diabetes, ulcers, inflammation, cancer, and cardiovascular diseases. Interestingly, many essential oils from Tajikistan show remarkable differences in their compositions compared to samples from other geographical locations, which can be attributed to the very different climatic and topographic characteristics of the country. Future studies on ecological, phytochemical, and therapeutic characteristics of medicinal plants from Tajikistan, in comparison to other locations outside and within the country, are encouraged. In addition, we encourage the preservation of traditional knowledge and uses of medicinal plants and their conservation.

## References

[1] Wink M., van Wyk B.E. (2008). Mind-Altering and Poisonous Plants of the World.

[2] Van Wyk B.E., Wink M. (2004). Medicinal Plants of the World.

[3] Rahmonov O., Majgier L., Andrejczuk W., Banaszek J., Karkosz D., Parusel T., Szymczyk A. (2013). Landscape diversity and biodiversity of Fann Mountains (Tajikistan). Ekológia (Bratislava).

[4] Nowak A., Nowak S., Nobis M., Nobis A. (2014). Vegetation of rock clefts and ledges in the Pamir Alai Mts, Tajikistan (Middle Asia). Cent. Eur. J. Biol..

[5] Vanselow K.A. (2011). The High-Mountain Pastures of the Eastern Pamirs (Tajikistan). An Evaluation of the Ecological Basis and the Pasture Potential. Ph.D. Thesis.

[6] Eisenman S.W., Zaurov D.E., Struwe L. (2013). Medicinal Plants of Central Asia: Uzbekistan and Kyrgyzstan.

[7] Nuraliev Y.N. (2008). Phytotherapy in Tajik traditional medicine and its perspective for modern medicine. The Problems of Phytotherapy and Phytopharmacology.

[8] Wink M. (2008). Evolutionary advantage and molecular modes of action of multi-component mixtures used in phytomedicine. Curr. Drug Metabol..

[9] Kholnazarov B.M. (2004). Razrabotka i issledovanie mazi iz efirnogo masla dushizi melkozvetkovoy na osnove bentonita (Investigation of Ointment of Oregano Oil on the Base of Bentonite). Ph.D. Thesis.

[10] Mabberley D.J. (2008). Mabberley’s Plant Book.

[11] Hojimatov M. (1989). Dikorastushie lekarstvennie rasteniya Tadjikistana.

[12] Kurbanov B. (1992). Lekarstvennie rasteniya—Pomoshnik cheloveka.

[13] Williams K. (2012). Medicinal plants in Tajikistan: An alternative livelihood option. In Proceedings of the X International People-Plant Symposium on Digging Deeper: Approaches to Research in Horticultural Therapy and Therapeutic Horticulture, Truro, NS, Canada, 5–8 August 2010. Acta Horticulturae.

[14] Nuraliev Y.N. (1989). Lekarstvennie Rasteniya.

[15] Kiyanpour V., Fakhari A., Asghari B., Yousefzadi M. (2011). Chemical composition and antibacterial activity of the essential oil of *Achillea filipendulina* (Asteraceae). Planta Med..

[16] Sharopov F.S., Setzer W.N. (2010). Composition of the essential oil of *Achillea filipendulina* Lam. from Tajikistan. Der Pharm. Chem..

[17] Harris B., Baser H.K.C., Buchbauer G. (2010). Phytotherapeutic uses of essential oils. Handbook of Essential Oils: Science, Technology, and Applications.

[18] Valant-Vetschera K.M., Wollenweber E. (1996). Comparative analysis of leaf exudate flavonoids in *Achillea* subsect. Filipendulinae. Biochem. System. Ecol..

[19] Ono K., Nakane H., Fukushima M., Chermann J.C., Barré-Sinoussi F. (1990). Differential inhibitory effects of various flavonoids on the activities of reverse transiptase and cellular DNA and RNA polymerases. Eur. J. Biochem..

[20] Farnet C.M., Wang B., Lipford J.R., Bushman F.D. (1996). Differential inhibition of HIV-1 preintegration complexes and purified integrase protein by small molecules. Proc. Natl. Acad. Sci. USA.

[21] Beutler J.A., Cardellina J.H., Lin C.M., Hamel E., Cragg G.M., Boyd M.R. (1993). Centaureidin, a cytotoxic flavone from *Polymnia fruticosa*, inhibits tubulin polymerization. Bioorg. Med. Chem. Lett..

[22] Sharopov F.S., Wink M., Gulmurodov I.S., Isupov S.J., Zhang H., Setzer W.N. (2013). Composition and bioactivity of the essential oil of *Anethum graveolens* L. from Tajikistan. Int. J. Med. Aromat. Plants.

[23] Yu Z., Wang W., Xu L., Dong J., Jing Y. (2008). *d*-Limonene and *d*-carvone induce apoptosis in HL-60 cells through activation of caspase-8. Asian J. Tradit. Med..

[24] Jäger W., Baser H.K.C., Buchbauer G. (2010). Metabolism of terpenoids in animal models and humans. Handbook of Essential Oils: Science, Technology, and Applications.

[25] Pelkonen O., Abass K., Wiesner J. (2013). Thujone and thujone-containing herbal medicinal and botanical products: Toxicological assessment. Regul. Toxicol. Pharmacol..

[26] Sharopov F.S., Sulaimonova V.A., Setzer W.N. (2012). Composition of the essential oil of *Artemisia absinthium* from Tajikistan. Rec. Nat. Prod..

[27] Sharopov F.S., Setzer W.N. (2011). Thujone-rich essential oils of *Artemisia rutifolia* Stephan ex Spreng. growing wild in Tajikistan. J. Essent. Oil Bear. Plants.

[28] Deiml T., Haseneder R., Zieglgansberger W., Rammes G., Eisensamer B., Rupprecht R., Hapfelmeier G. (2004). α-Thujone reduces 5-HT_3_ receptor activity by an effect on the agonist-induced desensitization. Neuropharmacology.

[29] Jakupovic J., Tan R.X., Bohlmann F., Jia Z.J., Huneck S. (1991). Sesquiterpene lactones from *Artemisia rutifolia*. Phytochemistry.

[30] Tan R.X., Jia Z.J., Jakupovic J., Bohlmann F., Huneck S. (1991). Sesquiterpene lactones from *Artemisia rutifolia*. Phytochemistry.

[31] Tan R.X., Jia Z.J. (1992). Sesquiterpenes from *Artemisia rutifolia*. Phytochemistry.

[32] Simonsen H.T., Weitzel C., Christensen S.B., Ramawat K.G., Méreillon J.M. (2013). Guaianolide sesquiterpenoids: Pharmacology and biosynthesis. Natural Products.

[33] Maries R.J., Pazos-Sanou L., Compadre C.M., Pezzuto J.M., Bloszyk E., Arnason J.T. (1995). Sesquiterpene lactones revisited. Rec. Adv. Phytochem..

[34] Sharopov F.S., Setzer W.N. (2011). The essential oil of *Artemesia scoparia* from Tajikistan is dominated by phenylacetylenes. Nat. Prod. Commun..

[35] Christensen L.P., Brandt K. (2006). Bioactive polyacetylenes in food plants of the Apiaceae family: Occurrence, bioactivity and analysis. J. Pharm. Biomed. Anal..

[36] Silva A.C.R.D., Lopes P.M., Azevedo M.M.B.D., Costa D.C.M., Alviano C.S., Alviano D.S. (2012). Biological activities of α-pinene and β-pinene enantiomers. Molecules.

[37] Sakhobiddinov S.S. (1948). Dikorastushie lekarstvennie rasteniya Sredney Azii. Gosizdat UzSSR, Tashkent.

[38] Jalilzadeh-Amin G., Maham M., Dalir-Naghadeh B., Kheiri F. (2011). Effects of *Bunium persicum* (Boiss.) essential oil on the contractile responses of smooth muscle (an *in vitro* study). Vet. Res. Forum.

[39] Céspedes C.L., Céspedes C.L., Sampietro D.A., Seigler D.S., Rai M. (2013). Antioxidant and biocidal activities from natural sources: An overview. Natural Antioxidants and Biocides from Wild Medicinal Plants.

[40] Reichling J., Wink M. (2010). Plant–microbe interactions and secondary metabolites with antibacterial, antifungal and antiviral properties. Annual Plant Reviews: Functions and Biotechnology of Plant Secondary Metabolites.

[41] Helander I.M., Alakomi H.L., Latva-Kala K., Mattila-Sandholm T., Pol I., Smid E.J., Gorris L.G.M., von Wright A. (1998). Characterization of the action of selected essential oil components on Gram-negative bacteria. J. Agric. Food Chem..

[42] Cantore P.L., Shanmugaiah V., Iacobellis N.S. (2009). Antibacterial activity of essential oil components and their potential use in seed disinfection. J. Agric. Food Chem..

[43] Sekine T., Sugano M., Majid A., Fujii Y. (2007). Antifungal effects of volatile compounds from black zira (*Bunium persicum*) and other spices and herbs. J. Chem. Ecol..

[44] Dudchenko L.G., Kozyakov A.S., Krivenko V.V. (1989). Priyno-aromaticheskie i pryano-vkusovie rasteniya.

[45] Sharopov F.S., Wink M., Khalifaev D.R., Zhang H., Dosoky N.S., Setzer W.N. (2013). Chemical composition and antiproliferative activity of the essential oil of *Galagania fragrantissima* Lipsky (Apiaceae). Am. J. Essent. Oil Nat. Prod..

[46] Rodríguez-Landa J.F., Contreras C.M. (2003). A review of clinical and experimental observations about antidepressant actions and side effects produced by *Hypericum perforatum* extracts. Phytomedicine.

[47] Mennini T., Gobbi M. (2004). The antidepressant mechanism of *Hypericum perforatum*. Life Sci..

[48] Butterweck V., Petereit F., Winterhoff H., Nahrstedt A. (1998). Solubilized hypericin and pseudohypericin from *Hypericum perforatum* exert antidepressant activity in the forced swimming test. Planta Med..

[49] Müller W.E., Singer A., Wonnemann M. (2001). Hyperforin—Antidepressant activity by a novel mechanism of action. Pharmacopsychiatry.

[50] Bruneton J. (1999). Pharmacognosy, Phytochemistry, Medicinal Plants.

[51] Yesilada E., Honda G., Sezik E., Tabata M., Fujita T., Tanaka T., Takeda Y., Takaishi Y. (1995). Traditional medicine in Turkey. V. Folk medicine in the inner Taurus Mountains. J. Ethnopharmacol..

[52] Süntar I.P., Akkol E.K., Yılmazer D., Baykal T., Kırmızıbekmez H., Alper M., Yeşilada E. (2010). Investigations on the *in vivo* wound healing potential of *Hypericum perforatum* L. J. Ethnopharmacol..

[53] Dost T., Ozkayran H., Gokalp F., Yenisey C., Birincioglu M. (2009). The effect of *Hypericum perforatum* (St. John’s wort) on experimental colitis in rat. Dig. Dis. Sci..

[54] Mozaffari S., Esmaily H., Rahimi R., Baeeri M., Sanei Y., Asadi-Shahmirzadi A., Salehi-Sumaghi M.H., Abdollahi M. (2011). Effects of *Hypericum perforatum* extract on rat irritable bowel syndrome. Pharmacogn. Mag..

[55] Sharopov F.S., Gulmurodov I.S., Setzer W.N. (2010). Essential oil composition of *Hypericum perforatum* L. and *Hypericum scabrum* L. growing wild in Tajikistan. J. Chem. Pharm. Res..

[56] Schmidt J.M., Noletto J.A., Vogler B., Setzer W.N. (2006). Abaco bush medicine: Chemical composition of the essential oils of four aromatic medicinal plants from Abaco Island, Bahamas. J. Herbs Spices Med. Plants.

[57] Legault J., Pichette A. (2007). Potentiating effect of β-caryophyllene on anticancer activity of α-humulene, isocaryophyllene and paclitaxel. J. Pharm. Pharmacol..

[58] Kızıl G., Toger Z., Özen H.Ç., Aytekin Ç. (2004). The antimicrobial activity of essential oils of *Hypericum scabrum*, *Hypericum scabroides* and *Hypericum triquetrifolium*. Phytother. Res..

[59] Erdoğrul Ö., Azirak S., Tosyali C. (2004). Antimicrobial activities of *Hypericum scabrum* L. extracts. KSU J. Sci. Eng..

[60] Rufino A.T., Ribeiro M., Judas F., Salgueiro L., Lopes M.C., Cavaleiro C., Mendes A.F. (2014). Anti-inflammatory and chondroprotective activity of (+)-α-pinene: Structural and enantiomeric selectivity. J. Nat. Prod..

[61] Ibn Sina A.A. (1982). The Canon of Medicine (Канон врачебной науки).

[62] Sharopov F.S., Kukaniev M.A., Thompson R.M., Satyal P., Setzer W.N. (2012). Composition and antimicrobial activity of the essential oil of *Hyssopus seravschanicus* growing wild in Tajikistan. Der Pharm. Chem..

[63] Kizil S., Toncer O., Ipek A., Arslan N., Saglam S., Khawar K.M. (2008). Blooming stages of Turkish hyssop (*Hyssopus officinalis* L.) affect essential oil composition. Acta Agric. Scand. B.

[64] Cvijovic M., Djukic D., Mandic L., Acamovic-Djokovic G., Pesakovic M. (2010). Composition and antimicrobial activity of essential oils of some medicinal and spice plants. Chem. Nat. Comp..

[65] Moradkhani H., Sargsyan E., Bibak H., Naseri B., Sadat-Hosseini M., Fayazi-Barjin A., Meftahizade H. (2010). *Mellisa officinali**s* L., a valuable medicinal plant: A review. J. Med. Plants Res..

[66] Setzer W.N. (2009). Essential oils and anxiolytic aromatherapy. Nat. Prod. Commun..

[67] Sorensen J.M. (2000). Melissa officinalis. Int. J. Aromather..

[68] Sharopov F.S., Wink M., Khalifaev D.R., Zhang H., Dosoky N.S., Setzer W.N. (2013). Composition and bioactivity of the essential oil of *Melissa officinalis* L. growing wild in Tajikistan. Int. J. Tradit. Nat. Med..

[69] Wright B.S., Bansal A., Moriarity D.M., Takaku S., Setzer W.N. (2007). Cytotoxic leaf essential oils from Neotropical Lauraceae: Synergistic effects of essential oil components. Nat. Prod. Commun..

[70] Sadraei H., Ghannadi A., Malekshahi K. (2003). Relaxant effect of essential oil of *Melissa officinalis* and citral on rat ileum contractions. Fitoterapia.

[71] Azarmi Y.A., Atefeh M., Hossein B. (2009). Role of endothelium on relaxant effect of geraniol in isolated rat aorta. Pharmaceut. Sci..

[72] Makhlayuk V.P. (1967). Lekarstvennie rasteniya v Narodnoy Medicine (Medicinal Plants in Folk Medicine).

[73] Sharopov F.S., Sulaimonova V.A., Setzer W.N. (2012). Essential oil composition of *Mentha longifolia* from wild populations growing in Tajikistan. J. Med. Active Plants.

[74] Viljoen A.M., Petkar S., van Vuuren S.F., Figueiredo A.C., Pedro L.G., Barroso J.G. (2006). The chemo-geographical variation in essential oil composition and the antimicrobial properties of “wild mint”—*Mentha longifolia* subsp. *polyadena* (Lamiaceae) in southern Africa. J. Essent. Oil Res..

[75] Lachance S., Grange G. (2014). Repellent effectiveness of seven plant essential oils, sunflower oil and natural insecticides against horn flies on pastured dairy cows and heifers. Med. Vet. Entomol..

[76] Amer A., Mehlhorn H. (2006). Repellency effect of forty-one essential oils against *Aedes*, *Anopheles*, and *Culex* mosquitoes. Parasitol. Res..

[77] López M.D., Jordán M.J., Pascual-Villalobos M.J. (2008). Toxic compounds in essential oils of coriander, caraway and basil active against stored rice pests. J. Stored Prod. Res..

[78] Moretti M.D.L., Peana A.T., Satta M. (1997). A study on anti-inflammatory and peripheral analgesic action of *Salvia sclarea* oil and its main components. J. Essent. Oil Res..

[79] Bassolé I.H.N., Lamien-Meda A., Bayala B., Tirogo S., Franz C., Novak J., Nebié R.C., Dicko M.H. (2010). Composition and antimicrobial activities of *Lipp**ia multiflora* Moldenke, *Mentha* x *piperita* L. and *Ocimum basilicum* L. essential oils and their major monoterpene alcohols alone and in combination. Molecules.

[80] Chiang L.C., Ng L.T., Cheng P.W., Chiang W., Lin C.C. (2005). Antiviral activities of extracts and selected pure constituents of *Ocimum basilicum*. Clin. Exp. Pharmacol. Physiol..

[81] Denisenko P.P., Nuraliev Y.N., Zubaidova T.M., Nuraliev Y.N. (2008). Pharmacology of herb *Origanum tyttanthum*. The Problems of Phytotherapy and Phytopharmacology.

[82] Jurbi O.B. (1988). Lekarstvennie rasteniya SSSR.

[83] Sharopov F.S., Kukaniev M.A., Setzer W.N. (2011). Composition of the essential oil of *Origanum tyttanthum* from Tajikistan. Nat. Prod. Commun..

[84] Wink M., Wink M. (2010). Biochemistry, physiology and ecological functions of secondary metabolites. Annual Plant Reviews, Vol. 40. Biochemistry of Plant Secondary Metabolism.

[85] Baričevič D., Bartol T., Kintzios S.E. (2002). The biological/pharmacological activity of the *Origanum* genus. Oregano. The Genera Origanum and Lippia.

[86] Zhang H. (2013). A Gas-Chromatographic/Mass Spectral Analysis of Aromatic Medicinal Plants from Tajikistan. Master’s Thesis.

[87] Sharopov F.S., Zhang H., Setzer W.N. (2014). Composition of geranium (*Pelargonium graveolens*) essential oil from Tajikistan. Am. J. Essent. Oil Nat. Prod..

[88] Sharopov F.S., Setzer W.N. (2012). The essential oil of *Salvia sclarea* L. from Tajikistan. Rec. Nat. Prod..

[89] Heptinstall S., Awang D.V.C., Dawson B.A., Kindack D., Knight D.W., May J. (1992). Parthenolide content and bioactivity of feverfew (*Tanacetum parthenium* (L.) Schultz-Bip.). Estimation of commercial and authenticated feverfew products. J. Pharm. Pharmacol..

[90] Williams C.A., Hoult J.R.S., Harborne J.B., Greenham J., Eagles J. (1995). A biologically active lipophilic flavonol from *Tanacetum part**henium*. Phytochemistry.

[91] Sharopov F.S., Setzer W.N., Isupov S.J., Wink M. (2015). Composition and bioactivity of the essential oil of *Tanacetum parthenium* from a wild population growing in Tajikistan. Am. J. Essent. Oils Nat. Prod..

[92] Sharopov F.S., Setzer W.N. (2011). Chemical diversity of *Ziziphora clinopodioides*: Composition of the essential oil of *Z. clinopodioides* from Tajikistan. Nat. Prod. Commun..

[93] Primo V., Rovera M., Zanon S., Oliva M., Demo M., Daghero J., Sabini L. (2001). Determinación de la actividad antibacteriana y antiviral del aceite esencial de *Minthostachys verticillata* (Griseb.) Epling. Rev. Argent. Microbiol..

